# A municipality-specific analysis to investigate persistent increased incidence rates of childhood leukaemia near the nuclear power plant of Krümmel in Germany

**DOI:** 10.1007/s10654-024-01182-w

**Published:** 2024-11-26

**Authors:** Emilio Gianicolo, Antonello Russo, Rossana Di Staso, Cécile M. Ronckers, Irene Schmidtmann, Daniel Wollschläger, Maria Blettner

**Affiliations:** 1 Institute of Medical Biostatistics, Epidemiology and Informatics (IMBEI), University Medical Center of the Johannes Gutenberg University of Mainz, Mainz, Germany; 2 National Research Council, Institute of Clinical Physiology, Lecce, Italy; 3 Independent Researcher, Lecce, Italy; 4 University of Bologna, Bologna, Italy

**Keywords:** Nnuclear power plant, Childhood leukaemia, Krümmel, Germany, Reference, Population, Poisson-Gamma models

## Abstract

Increased incidence rates for childhood leukaemia have been reported in municipalities close to the nuclear power plant (NPP) Krümmel (Geesthacht, Germany). Methodological challenges arise when analysing this association at ecological level. They include the use of an appropriate reference population, unstable estimates of standardised incidence ratios (SIRs), and the potential role of prevailing winds. The aim of our study is to address these challenges. The German Childhood Cancer Registry provided data on leukaemia in children under 15 years (2004–2019). The German Federal Statistical Office provided the population data. The study region included all municipalities with ≥ 75% surface area within 50 kms from the Krümmel NPP. We calculated SIRs using national and regional reference rates. Smoothed incidence relative rates (IRRs) were calculated and mapped to check for potential patterns associated with prevailing winds. Overall 356 cases of childhood leukaemia were observed in the study region (321 municipalities) during 2004–2019. SIRs based on national reference rates show nearly no difference to those calculated using the regional rates as reference. Increased SIR and IRR were observed in Geesthacht (observed-cases = eight; SIR = 2.29; 95% confidence interval: 0.99–4.51. IRR = 1.80; 95% credibility interval: 0.88–2.79). The analysis of the IRR map does not show patterns associated with prevailing winds. Using a regional population as the reference, we found evidence that there may still be an increased risk for childhood leukaemia in Geesthacht. However, IRR estimates are uncertain and credibility intervals are compatible with the absence of elevated risk. The persistent evidence of risk of childhood leukaemia in Geesthacht warrants further epidemiological surveillance.

**Supplementary Information:**

The online version contains supplementary material available at 10.1007/s10654-024-01182-w.

## Introduction

Since the beginning of the 1980s, several authors in different countries have investigated the association between childhood leukaemia and residential proximity to nuclear power plants (NPPs) with inconsistent results. In the early 1980s, concerns about the potential health effects of radiation exposure from NPPs became widespread among the British population. The issue hit the headlines with a television documentary entitled “Windscale the Nuclear Laundry” [1]. The documentary referred to anecdotal evidence of increased leukaemia risk in children in the coastal town of Seascale, adjacent to the Sellafield nuclear site also involved in nuclear waste processing. Subsequently, in addition to several reports from the Committee on Medical Aspects of Radiation in the Environment (COMARE) [2], epidemiological studies were published on the population near Sellafield and other NPPs in the UK [3]. A persistent increased risk of leukaemia was reported in under 15-year-olds children resident around the nuclear installations of Sellafield and Dounreay (UK) from 1963 to 1990. These results were not confirmed in more recent periods.

Several articles addressed the association between incidence of childhood leukaemia and residential distance to NPPs since the 1990s (e. g. [4, 5]) including those focused on the NPP in Krümmel, located in the urban district of Geesthacht (Germany). The NPP in Krümmel was built in the 1970s. Power operations started in 1984 and stopped in 2007. It was officially closed in 2011. The NPP in Krümmel is near to the Nuclear Research Centre GKSS (Gesellschaft für Kernenergieverwertung in Schiffbau und Schiffahrt). According to the German Federal Office for Radiation Protection, the estimated annual radiation dose for children living near the NPP in Krümmel was < 2 µSv/a (doses to the thyroid < 3 µSv/a) recorded until 2010 and < 0.1 µSv/a thereafter [6].

In the late 1990s, increased incidence rates vs. the national reference were reported among children residing in several municipalities within ten kilometres from the NPP in Krümmel. Further publications confirmed this observation in the following years (e. g. [7]), including recently with data up to 2019 [8, 9].

Most of these publications are based on ecological, i.e. region-based aggregate data without information on individual radiation exposure or other potential risk factors. Grosche et al. reported on several potential risk factors that have been investigated in this area, including indoor risk factors (radon and drinking water), air pollutants, other forms of ionising radiation, and electro-magnetic fields [4]. They concluded that the cause for the increased risk of childhood leukaemia near the NPP in Krümmel “has to be considered as unknown” [4].

Several methodological challenges arise when analysing the association of radiation exposure and leukaemia incidence at an ecological level.

Calculating incidence rate ratios requires cancer rates from a suitable reference population. One may argue that a population closer to the NPP is more representative regarding potential confounding factors or effect modifiers as compared to the full national population. Hence, incidence rates observed in the study population are supposed to be the same as those observed in the reference population (counterfactual scenario), unless a risk factor causes a change in the risk profile among persons living in the study area. Thus, the population under study and the reference population ought to be as similar as possible and only differ according to the exposure variable under study.

A second issue is the rarity of the outcome under study, which leads to low statistical power and unstable estimates of standardised incidence ratios [10].

A third issue is the potential role of meteorological factors. In the published ecological studies, the distance to the NPP was used as a surrogate for potential radiation exposure. However, it has been hypothesised that an increased risk of radiation exposure must be expected among populations on the windward side of power plants as airborne radioactive particles emanating from the NPP would be carried away by the wind. In Geesthacht, the prevailing wind direction is from western sectors. Therefore, it was argued, radiation exposure on the eastern side of an NPP should be higher than on the western side [11]. If airborne releases of radioactivity from an NPP were a true risk factor, increased incidence cancer rates are to be expected mainly in the downwind direction. Along those lines, increased incidence rates have to be expected also farther away, i. e. more than ten kilometres from NPPs.

The aim of our study is to separately address these three major challenges. To do this, we performed a municipality-specific analysis using (a) a regional reference population in place of a national one, (b) a Bayesian approach in order to obtain more stable risk estimates, and finally, (c) employing a disease mapping approach to evaluate spatial patterns potentially associated with wind directions and tested for spatial correlation.

## Material and methods

The study region included all municipalities with at least 75% of their surface area within 50 kms from the NPP in Krümmel.

The German Childhood Cancer Registry provided data on leukaemia incidence in children under 15 years of age for the years 2004–2019 by municipality of residence at the time of diagnosis. The German Federal Statistical Office provided the corresponding population data.

For the time period 2004–2019, we calculated age-standardised incidence ratios (SIRs) and associated 95% confidence intervals. The age groups 0, 1–4, 5–9, and 10–14 years and single calendar years were used for standardisation. We used two reference populations: the complete German population as well as the population of federal States with at least one municipality within 50 km from the NPP in Krümmel: Schleswig–Holstein, Lower Saxony, Hamburg, and Mecklenburg-Western Pomerania. SIRs were calculated using PROC STDRATE in SAS V9.4, requesting confidence intervals based on a Poisson distribution.

To overcome the issue of unstable estimates for small municipalities, smoothed incidence relative rates (IRRs) and associated 95% credibility intervals were calculated using a two level hierarchical Bayesian Poisson-gamma model [12]. At the first level, the counts ${y}_{i}$ of childhood leukaemia in the municipality i of the study region are assumed to follow a Poisson distribution with parameter λ, whereby λ is a function of person years n at risk and the incidence rate of childhood leukaemia θ in each municipality.

$${y}_{i}$$~ Poisson ($${\lambda }_{i}$$). Where:

${y}_{i}$, with *i* = 1 …m are counts of childhood leukaemia cases in each municipality of the study region and ${\lambda }_{i}= {n}_{i}\times {\theta }_{i}$.

On the second level, we assumed that the ${\theta }_{i}$ follow a Gamma distribution with parameters α and β:

${\theta }_{i}$ ~ Gamma(α,β). We fixed both parameters following Lawson [13].

With this method, the more unstable the municipality-specific estimates are, the larger the adjustment from smoothing is. In other words, the estimates are smoothed towards the overall mean and allow for modelling Poisson over-dispersion [10]. In this context, 10,000 iterations of Markov Chain Monte Carlo (MCMC) sampling were used. Convergence was visually checked assessing the trace plot to inspect sampling behaviour. From this evaluation, we decided to discard the first 2000 values as “burn-in”, since sampling showed relatively constant mean and variance for the remaining iterations. In addition, autocorrelation of MCMC runs was visually assessed by plotting the autocorrelation function [14]. Smoothed incidence relative rates were calculated using the WinBUGS V1.4 software. Finally, using the QGIS software we mapped the smoothed incidence relative rates calculated for each community in order to visually check for potential patterns associated with the wind direction prevailing in the region under study, i. e. from west to east. We used Tango’s index to detect spatial clustering [15] and used the R-package *DCluster* for this purpose [16]. This index proposed by Tango in 1995 is a method for global clustering evaluation (see supplementary material for further details) [17]. A total number of 999 non-parametric bootstrap replicates were used to compute the p-value of the test.

## Results

Overall, 356 cases of childhood leukaemia were observed in 321 municipalities within a radius of 50 kms from the NPP in Krümmel from 2004 to 2019 (person years contributed up to age 15 years: 6,349,938). SIRs calculated using Germany as the reference population (SIR-GER) show nearly no difference compared to the SIRs calculated using the four adjacent federal States as a reference (SIR-4FS) (Table S1 in Supplementary Material). Overall, in the study area we observed a SIR-GER = 1.06 (95% confidence interval: 0.95–1.17) and a SIR-4FS = 1.03 (95% confidence interval: 0.93–1.15).

Municipality specific analysis showed that in Geesthacht, the SIR calculated using the four adjacent federal states as the reference is slightly lower than the SIR calculated using Germany as the reference (observed = 8 cases; person-years = 65,394; SIR-GER = 2.36; 95% confidence interval: 1.02–4.64; SIR-4FS = 2.29; 95% confidence interval: 0.99–4.51) (Table S1).

**Fig. 1:**
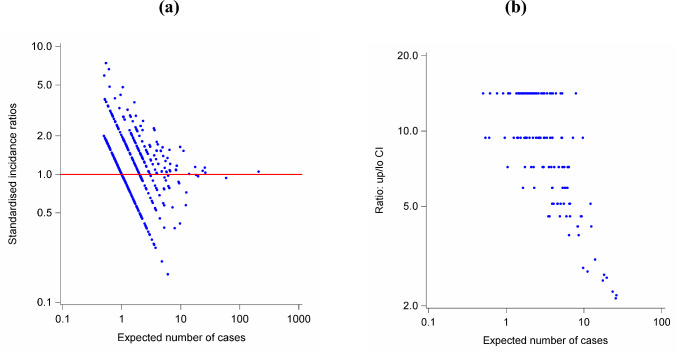
**a** scatter plot of SIRs on expected incidence cases in the municipalities considered in the analysis and **b** scatter plot of the ratio between upper and lower value of the 95% confidence interval. The log scale is used for both axis

Municipality-specific SIRs showed high variation at a low number of expected cases (Fig. 1, panel (a) and (b)). In fact, the increasing number of expected cases is associated with decreasing SIRs (Fig. 1, panel (a)) as well as decreasing ratios of the upper/lower values of the associated 95% confidence intervals (Fig. 1, panel (b)).

For some municipalities including Geesthacht smoothed relative rates are still higher than those of the reference population (IRR-Geesthacht = 1.80; 95% credibility interval: 0.88–3.03) (Table S1). Note that using the hierarchical Bayesian Poisson-gamma method all credibility intervals reported in Table S1 include 1.0.

**Fig. 2:**
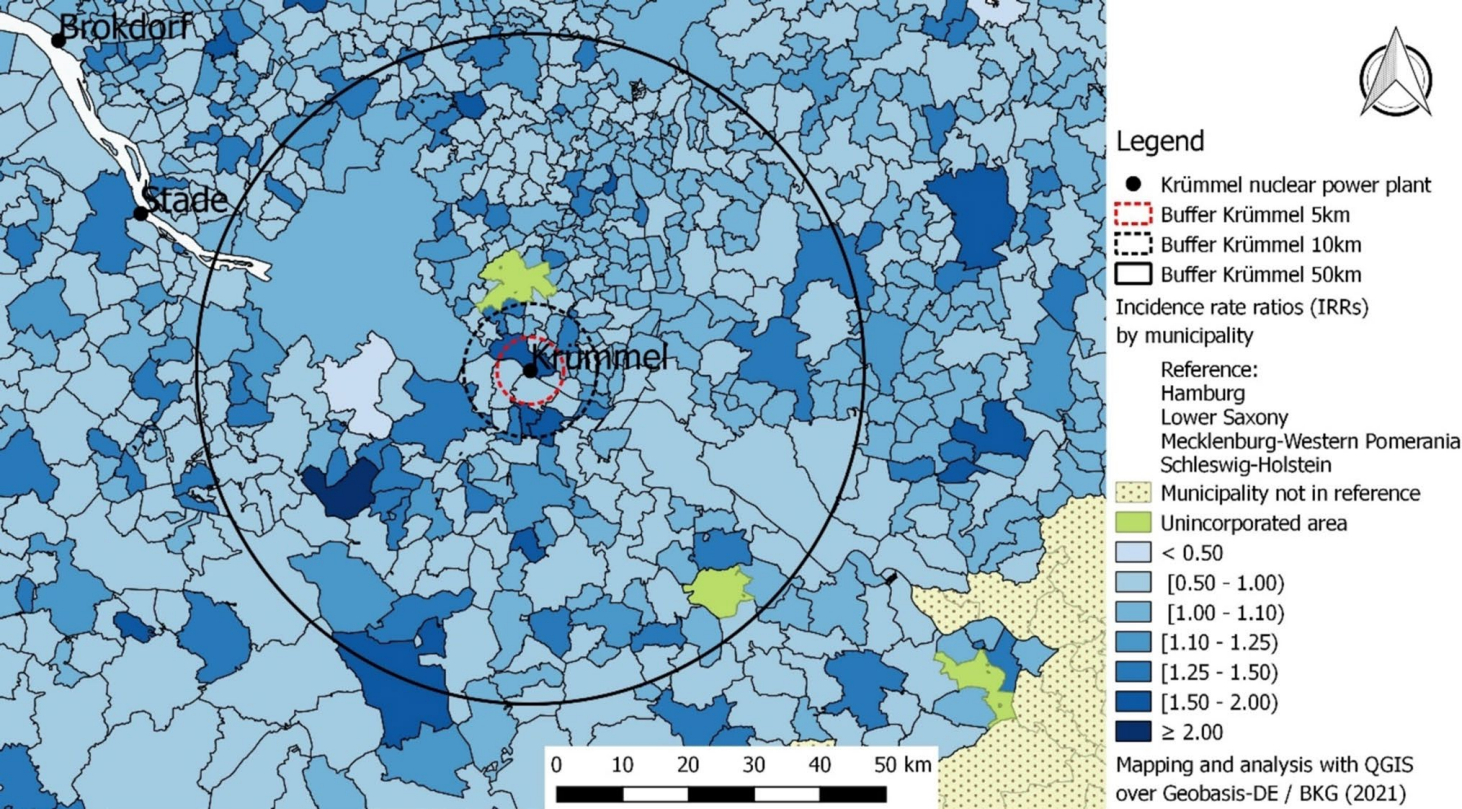
Maps of the incidence rate ratios (IRRs) resulting from the hierarchical Bayesian Poisson-Gamma method

The visual analysis of the maps reporting IRRs shows some heterogeneity, which disappears when looking at credibility intervals, which regularly overlap (Table S1 in Supplementary Material). Furthermore, visually no pattern associated with prevailing wind directions is observed (Fig. 2). Accordingly, the Tango index did not show any evidence of spatial correlation (Tango index = 0.01; *p*-value = 0.47).

## Discussion and conclusion

Using a regional population as the reference in place of the national one, we found increased SIRs for childhood leukaemia in Geesthacht for the observation period 2004–2019. Smoothed IRR calculated after accounting for over-dispersion are characterised by narrower credibility intervals than the SIRs’ confidence intervals but are consistent with absence of risk.

IRRs do not show any pattern associated with prevailing wind direction. Accordingly, analysis accounting for spatial correlation might not be warranted.

Multidimensional disease mapping with hypothesising space and time risk interaction is used in different settings including cancer risk associated to several environmental risk factors. However, because of small numbers we did not stratify our analysis according to year of diagnosis.

Ecological regression to test the role of the wind while accounting for covariates (e. g. socioeconomic status) can be an option in this kind of study. However, because there is no evidence of spatial correlation and no evidence of even a moderate directional trend in our study, we believe that due to small numbers such an analysis would not be supported by sufficient statistical power.

A limitation of our work is that we only have ecological data. We aimed to improve on the analyses published so far while recognising that the most important issues i. e. lack of individual exposure and the low number of events of interest cannot be solved by our approach.

Some of the municipalities within 50 km from the NPP in Krümmel also fall within a 50 km-radius from two other NPPs: Stade, which went out of active service on 14 November 2003, and Brockdorf, which went out of active service on 31 December 2021.A specific analysis on these municipalities was outside the scope of our work.

In comparison to previously published studies covering the same study-period [8, 9], the present study considers single municipalities as statistical units.

To the best of our knowledge this is the first study using a Bayesian approach to analyse the incidence of leukaemia among children resident in this area. A Bayesian approach based on random effects models was also used by Hoffman and Schlattmann to analyse the geographical distribution of leukaemia in all ages among residents in three counties adjacent to Krümmel [18]. Hoffmann and Schlattmann extended their analysis to all ages and found an increased risk of chronic lymphatic leukaemia in males in the 5 km region around the plant. They did not find any trend with distance.

A local population of six adjacent counties was also used by Hoffmann et al. as the reference for the calculation of SIRs in addition to the German population [7]. As in our study, the authors found that the SIR was slightly lower using Germany as the reference.

After implementing methodological approaches to face issues that arose in the literature on the study of the association between childhood leukaemia and proximity to nuclear power plants, our results do not support the hypothesis of a geographical pattern due to the wind direction prevailing in the region around Krümmel nuclear power plant. Waterborne radioactivity is also possible. However, this investigation was also outside the aims of our study.

The increased risk observed in Geesthacht even after smoothing the estimates deserves epidemiological surveillance*.*

## Supplementary Information

Below is the link to the electronic supplementary material.

Supplementary file1 (DOCX 68 KB)
