# Supplementary material for: A municipality-specific analysis to investigate persistent increased incidence rates of childhood leukaemia near the nuclear power plant of Krümmel in Germany
Source: Eur J Epidemiol. 2024 Nov 26;39(12):1373–8. doi: 10.1007/s10654-024-01182-w (PMC11680643; doi:10.1007/s10654-024-01182-w)
Supplement: Supplementary file 1 — Supplementary file1 (DOCX 68 KB) [file 10654_2024_1182_MOESM1_ESM.docx]

**Supplementary Material**

**On the Tango index.**

The test statistic T can be described as:

$$T =\sum_{i=1}^{m} \sum_{j=1}^{m} {w_{ij}a}_{ij}\left( r_{i}- p_{i} \right)$$

Where $r_{i} = \frac{y_{i}}{Y}$ and $p_{i} = \frac{n_{i}}{N}$.

$Y= \sum_{i=1}^{m} y_{i}$ and $S= \sum_{i=1}^{m} n_{i}$ are the sum of the total number of observed cases (Y) and the population size (N) in the municipality of the study region respectively.

Finally, $a_{ij}$ measure of closeness between the ith and the jth regions and $w_{ij}$ is a weight that can be assigned based on prior information. Under the null hypothesis $H_{0}:E\left( y_{i} \right)= \lambda s_{i}$ the total number of cases N in the region is a sufficient statistic for the parameter $\lambda$.

**Table S1 – Person-years, observed cases of leukaemia in children <15 years and expected cases calculated assuming Germany and four federal states as reference, standardised incidence rates (SIR) and incidence relative rates in municipalities with at least 75% of municipal area in a radius of 50 km from Krümmel nuclear power plant. 2004-2019.**

| Municipality | Person years (under 15 years of age) | Observed* | Reference population | | | | | | | | | | | IRR | 95% Confidence interval | | |
| --- | --- | --- | --- | --- | --- | --- | --- | --- | --- | --- | --- | --- | --- | --- | --- | --- | --- |
|  |  |  | Germany | | | | |  | Four federal states | | | | |  |  |  |  |
|  |  |  | Expected | SIR | 95% Confidence interval | | |  | Expected | SIR | 95% Confidence interval | | |  |  |  |  |
| **Study area** | **6,349,938** | **356** | **336.21** | **1.06** | **0.95** | **-** | **1.17** |  | **344.90** | **1.03** | **0.93** | **-** | **1.15** | **-** | **-** | **-** | **-** |
| Albsfelde | 140 | <5 |  | 0.00 |  | - |  |  |  | 0.00 |  | - | | 1.03 | 0.16 | - | 2.72 |
| Alt-Mölln | 1,864 | <5 |  | 10.76 |  | - |  |  |  | 10.37 |  | - | | 1.42 | 0.33 | - | 3.31 |
| Aumühle | 7,199 | <5 |  | 0.00 |  | - |  |  |  | 0.00 |  | - | | 0.89 | 0.13 | - | 2.33 |
| Bäk | 2,285 | <5 |  | 0.00 |  | - |  |  |  | 0.00 |  | - | | 0.98 | 0.15 | - | 2.61 |
| Bälau | 480 | <5 |  | 0.00 |  | - |  |  |  | 0.00 |  | - | | 1.02 | 0.16 | - | 2.71 |
| Basedow | 1,349 | <5 |  | 0.00 |  | - |  |  |  | 0.00 |  | - | | 1.00 | 0.15 | - | 2.63 |
| Basthorst | 874 | <5 |  | 0.00 |  | - |  |  |  | 0.00 |  | - | | 1.02 | 0.15 | - | 2.70 |
| Behlendorf | 1,084 | <5 |  | 0.00 |  | - |  |  |  | 0.00 |  | - | | 1.01 | 0.15 | - | 2.70 |
| Berkenthin | 5,897 | <5 |  | 0.00 |  | - |  |  |  | 0.00 |  | - | | 0.91 | 0.14 | - | 2.38 |
| Besenthal | 175 | <5 |  | 0.00 |  | - |  |  |  | 0.00 |  | - | | 1.04 | 0.15 | - | 2.73 |
| Bliestorf | 1,721 | <5 |  | 0.00 |  | - |  |  |  | 0.00 |  | - | | 0.99 | 0.14 | - | 2.64 |
| Börnsen | 11,125 | <5 |  | 0.00 |  | - |  |  |  | 0.00 |  | - | | 0.82 | 0.12 | - | 2.17 |
| Borstorf | 771 | <5 |  | 0.00 |  | - |  |  |  | 0.00 |  | - | | 1.02 | 0.15 | - | 2.73 |
| Breitenfelde | 4,701 | <5 |  | 0.00 |  | - |  |  |  | 0.00 |  | - | | 0.93 | 0.14 | - | 2.45 |
| Bröthen | 766 | <5 |  | 0.00 |  | - |  |  |  | 0.00 |  | - | | 1.02 | 0.15 | - | 2.69 |
| Brunsmark | 283 | <5 |  | 0.00 |  | - |  |  |  | 0.00 |  | - | | 1.03 | 0.15 | - | 2.73 |
| Brunstorf | 1,872 | <5 |  | 0.00 |  | - |  |  |  | 0.00 |  | - | | 0.99 | 0.14 | - | 2.63 |
| Buchholz | 702 | <5 |  | 0.00 |  | - |  |  |  | 0.00 |  | - | | 1.02 | 0.15 | - | 2.68 |
| Buchhorst | 248 | <5 |  | 0.00 |  | - |  |  |  | 0.00 |  | - | | 1.03 | 0.15 | - | 2.74 |
| Büchen | 12,897 | <5 |  | 0.00 |  | - |  |  |  | 0.00 |  | - | | 0.80 | 0.12 | - | 2.12 |
| Dahmker | 450 | <5 |  | 0.00 |  | - |  |  |  | 0.00 |  | - | | 1.03 | 0.16 | - | 2.69 |
| Dalldorf | 803 | <5 |  | 0.00 |  | - |  |  |  | 0.00 |  | - | | 1.03 | 0.16 | - | 2.70 |
| Dassendorf | 7,785 | <5 |  | 2.52 |  | - |  |  |  | 2.44 |  | - | | 1.26 | 0.28 | - | 2.95 |
| Düchelsdorf | 390 | <5 |  | 0.00 |  | - |  |  |  | 0.00 |  | - | | 1.02 | 0.15 | - | 2.69 |
| Duvensee | 1,440 | <5 |  | 0.00 |  | - |  |  |  | 0.00 |  | - | | 1.01 | 0.15 | - | 2.67 |
| Einhaus | 945 | <5 |  | 0.00 |  | - |  |  |  | 0.00 |  | - | | 1.02 | 0.15 | - | 2.69 |
| Elmenhorst | 2,233 | <5 |  | 0.00 |  | - |  |  |  | 0.00 |  | - | | 0.98 | 0.15 | - | 2.59 |
| Escheburg | 9,250 | <5 |  | 0.00 |  | - |  |  |  | 0.00 |  | - | | 0.85 | 0.13 | - | 2.24 |
| Fitzen | 724 | <5 |  | 0.00 |  | - |  |  |  | 0.00 |  | - | | 1.02 | 0.15 | - | 2.73 |
| Fredeburg | 110 | <5 |  | 0.00 |  | - |  |  |  | 0.00 |  | - | | 1.04 | 0.15 | - | 2.74 |
| Fuhlenhagen | 664 | <5 |  | 0.00 |  | - |  |  |  | 0.00 |  | - | | 1.03 | 0.15 | - | 2.71 |
| **Geesthacht** | **65,394** | **8** | **3.40** | **2.36** | **1.02** | **-** | **4.64** |  | **3.49** | **2.29** | **0.99** | **-** | **4.51** | **1.80** | **0.88** | **-** | **3.03** |
| Giesensdorf | 222 | <5 |  | 0.00 |  | - |  |  |  | 0.00 |  | - | | 1.03 | 0.16 | - | 2.74 |
| Göldenitz | 673 | <5 |  | 0.00 |  | - |  |  |  | 0.00 |  | - | | 1.02 | 0.15 | - | 2.69 |
| Göttin | 127 | <5 |  | 0.00 |  | - |  |  |  | 0.00 |  | - | | 1.04 | 0.16 | - | 2.76 |
| Grabau | 873 | <5 |  | 0.00 |  | - |  |  |  | 0.00 |  | - | | 1.03 | 0.15 | - | 2.77 |
| Grambek | 1,026 | <5 |  | 0.00 |  | - |  |  |  | 0.00 |  | - | | 1.02 | 0.15 | - | 2.65 |
| Grinau | 407 | <5 |  | 0.00 |  | - |  |  |  | 0.00 |  | - | | 1.03 | 0.15 | - | 2.71 |
| Groß Boden | 448 | <5 |  | 0.00 |  | - |  |  |  | 0.00 |  | - | | 1.03 | 0.15 | - | 2.72 |
| Groß Disnack | 140 | <5 |  | 0.00 |  | - |  |  |  | 0.00 |  | - | | 1.04 | 0.15 | - | 2.75 |
| Groß Pampau | 334 | <5 |  | 0.00 |  | - |  |  |  | 0.00 |  | - | | 1.03 | 0.16 | - | 2.73 |
| Groß Sarau | 2,552 | <5 |  | 0.00 |  | - |  |  |  | 0.00 |  | - | | 0.98 | 0.14 | - | 2.57 |
| Groß Schenkenberg | 1,487 | <5 |  | 0.00 |  | - |  |  |  | 0.00 |  | - | | 1.01 | 0.15 | - | 2.65 |
| Grove | 531 | <5 |  | 0.00 |  | - |  |  |  | 0.00 |  | - | | 1.03 | 0.15 | - | 2.70 |
| Gudow | 3,994 | <5 |  | 0.00 |  | - |  |  |  | 0.00 |  | - | | 0.95 | 0.14 | - | 2.50 |
| Gülzow | 2,812 | <5 |  | 7.05 |  | - |  |  |  | 6.80 |  | - | | 1.39 | 0.32 | - | 3.24 |
| Güster | 2,518 | <5 |  | 0.00 |  | - |  |  |  | 0.00 |  | - | | 0.98 | 0.15 | - | 2.60 |
| Hamfelde | 876 | <5 |  | 0.00 |  | - |  |  |  | 0.00 |  | - | | 1.02 | 0.15 | - | 2.68 |
| Hamwarde | 1,809 | <5 |  | 0.00 |  | - |  |  |  | 0.00 |  | - | | 1.00 | 0.15 | - | 2.61 |
| Harmsdorf | 742 | <5 |  | 0.00 |  | - |  |  |  | 0.00 |  | - | | 1.03 | 0.15 | - | 2.72 |
| Havekost | 345 | <5 |  | 0.00 |  | - |  |  |  | 0.00 |  | - | | 1.03 | 0.16 | - | 2.73 |
| Hohenhorn | 1,151 | <5 |  | 0.00 |  | - |  |  |  | 0.00 |  | - | | 1.00 | 0.15 | - | 2.67 |
| Hollenbek | 1,106 | <5 |  | 0.00 |  | - |  |  |  | 0.00 |  | - | | 1.01 | 0.16 | - | 2.69 |
| Hornbek | 367 | <5 |  | 0.00 |  | - |  |  |  | 0.00 |  | - | | 1.03 | 0.15 | - | 2.69 |
| Horst | 490 | <5 |  | 0.00 |  | - |  |  |  | 0.00 |  | - | | 1.03 | 0.15 | - | 2.68 |
| Juliusburg | 409 | <5 |  | 0.00 |  | - |  |  |  | 0.00 |  | - | | 1.04 | 0.15 | - | 2.77 |
| Kankelau | 468 | <5 |  | 0.00 |  | - |  |  |  | 0.00 |  | - | | 1.03 | 0.15 | - | 2.77 |
| Kasseburg | 1,063 | <5 |  | 0.00 |  | - |  |  |  | 0.00 |  | - | | 1.01 | 0.15 | - | 2.66 |
| Kastorf | 3,282 | <5 |  | 0.00 |  | - |  |  |  | 0.00 |  | - | | 0.97 | 0.15 | - | 2.57 |
| Kittlitz | 552 | <5 |  | 0.00 |  | - |  |  |  | 0.00 |  | - | | 1.04 | 0.15 | - | 2.69 |
| Klein Pampau | 1,368 | <5 |  | 0.00 |  | - |  |  |  | 0.00 |  | - | | 1.02 | 0.15 | - | 2.69 |
| Klein Zecher | 652 | <5 |  | 0.00 |  | - |  |  |  | 0.00 |  | - | | 1.02 | 0.15 | - | 2.68 |
| Klempau | 1,743 | <5 |  | 0.00 |  | - |  |  |  | 0.00 |  | - | | 1.00 | 0.15 | - | 2.65 |
| Klinkrade | 1,435 | <5 |  | 0.00 |  | - |  |  |  | 0.00 |  | - | | 1.01 | 0.15 | - | 2.65 |
| Koberg | 1,916 | <5 |  | 10.42 |  | - |  |  |  | 10.08 |  | - | | 1.43 | 0.34 | - | 3.34 |
| Köthel | 678 | <5 |  | 0.00 |  | - |  |  |  | 0.00 |  | - | | 1.03 | 0.15 | - | 2.74 |
| Kollow | 1,539 | <5 |  | 0.00 |  | - |  |  |  | 0.00 |  | - | | 1.01 | 0.15 | - | 2.61 |
| Kröppelshagen-Fahrendorf | 2,834 | <5 |  | 6.75 |  | - |  |  |  | 6.56 |  | - | | 1.39 | 0.32 | - | 3.25 |
| Krüzen | 962 | <5 |  | 0.00 |  | - |  |  |  | 0.00 |  | - | | 1.01 | 0.15 | - | 2.63 |
| Krukow | 273 | <5 |  | 0.00 |  | - |  |  |  | 0.00 |  | - | | 1.04 | 0.16 | - | 2.74 |
| Krummesse | 4,397 | <5 |  | 0.00 |  | - |  |  |  | 0.00 |  | - | | 0.95 | 0.13 | - | 2.52 |
| Kuddewörde | 3,286 | <5 |  | 0.00 |  | - |  |  |  | 0.00 |  | - | | 0.96 | 0.14 | - | 2.55 |
| Kühsen | 948 | <5 |  | 0.00 |  | - |  |  |  | 0.00 |  | - | | 1.02 | 0.15 | - | 2.67 |
| Kulpin | 435 | <5 |  | 0.00 |  | - |  |  |  | 0.00 |  | - | | 1.03 | 0.16 | - | 2.74 |
| Labenz | 2,279 | <5 |  | 0.00 |  | - |  |  |  | 0.00 |  | - | | 0.98 | 0.15 | - | 2.62 |
| Langenlehsten | 416 | <5 |  | 0.00 |  | - |  |  |  | 0.00 |  | - | | 1.03 | 0.16 | - | 2.68 |
| Lankau | 1,253 | <5 |  | 0.00 |  | - |  |  |  | 0.00 |  | - | | 1.01 | 0.15 | - | 2.65 |
| Lanze | 1,001 | <5 |  | 0.00 |  | - |  |  |  | 0.00 |  | - | | 1.02 | 0.15 | - | 2.69 |
| Lauenburg/Elbe | 26,290 | <5 |  | 1.48 |  | - |  |  |  | 1.44 |  | - | | 1.20 | 0.35 | - | 2.57 |
| Lehmrade | 1,142 | <5 |  | 0.00 |  | - |  |  |  | 0.00 |  | - | | 1.01 | 0.15 | - | 2.71 |
| Linau | 2,860 | <5 |  | 0.00 |  | - |  |  |  | 0.00 |  | - | | 0.97 | 0.14 | - | 2.53 |
| Lüchow | 738 | <5 |  | 0.00 |  | - |  |  |  | 0.00 |  | - | | 1.02 | 0.15 | - | 2.68 |
| Lütau | 1,844 | <5 |  | 0.00 |  | - |  |  |  | 0.00 |  | - | | 0.99 | 0.15 | - | 2.60 |
| Mechow | 303 | <5 |  | 0.00 |  | - |  |  |  | 0.00 |  | - | | 1.03 | 0.16 | - | 2.75 |
| Möhnsen | 1,390 | <5 |  | 14.43 |  | - |  |  |  | 14.08 |  | - | | 1.45 | 0.33 | - | 3.39 |
| Mölln | 38,519 | <5 |  | 0.51 |  | - |  |  |  | 0.49 |  | - | | 0.78 | 0.18 | - | 1.81 |
| Mühlenrade | 455 | <5 |  | 0.00 |  | - |  |  |  | 0.00 |  | - | | 1.02 | 0.15 | - | 2.70 |
| Müssen | 2,767 | <5 |  | 0.00 |  | - |  |  |  | 0.00 |  | - | | 0.97 | 0.15 | - | 2.54 |
| Mustin | 1,726 | <5 |  | 0.00 |  | - |  |  |  | 0.00 |  | - | | 0.99 | 0.15 | - | 2.60 |
| Niendorf bei Berkenthin | 454 | <5 |  | 0.00 |  | - |  |  |  | 0.00 |  | - | | 1.04 | 0.15 | - | 2.76 |
| Niendorf/Stecknitz | 1,784 | <5 |  | 0.00 |  | - |  |  |  | 0.00 |  | - | | 1.00 | 0.15 | - | 2.63 |
| Nusse | 3,144 | <5 |  | 12.70 |  | - |  |  |  | 12.25 |  | - | | 1.80 | 0.54 | - | 3.84 |
| Panten | 1,415 | <5 |  | 0.00 |  | - |  |  |  | 0.00 |  | - | | 1.00 | 0.15 | - | 2.65 |
| Pogeez | 1,014 | <5 |  | 0.00 |  | - |  |  |  | 0.00 |  | - | | 1.02 | 0.15 | - | 2.73 |
| Poggensee | 796 | <5 |  | 0.00 |  | - |  |  |  | 0.00 |  | - | | 1.02 | 0.15 | - | 2.70 |
| Ratzeburg | 31,914 | <5 |  | 0.00 |  | - |  |  |  | 0.00 |  | - | | 0.59 | 0.09 | - | 1.56 |
| Ritzerau | 669 | <5 |  | 0.00 |  | - |  |  |  | 0.00 |  | - | | 1.03 | 0.16 | - | 2.75 |
| Römnitz | 79 | <5 |  | 0.00 |  | - |  |  |  | 0.00 |  | - | | 1.04 | 0.16 | - | 2.76 |
| Rondeshagen | 2,235 | <5 |  | 0.00 |  | - |  |  |  | 0.00 |  | - | | 0.99 | 0.15 | - | 2.63 |
| Roseburg | 1,206 | <5 |  | 0.00 |  | - |  |  |  | 0.00 |  | - | | 1.01 | 0.15 | - | 2.63 |
| Sahms | 946 | <5 |  | 0.00 |  | - |  |  |  | 0.00 |  | - | | 1.02 | 0.15 | - | 2.69 |
| Salem | 1,190 | <5 |  | 0.00 |  | - |  |  |  | 0.00 |  | - | | 1.01 | 0.15 | - | 2.73 |
| Sandesneben | 5,379 | <5 |  | 0.00 |  | - |  |  |  | 0.00 |  | - | | 0.93 | 0.13 | - | 2.47 |
| Schiphorst | 1,761 | <5 |  | 0.00 |  | - |  |  |  | 0.00 |  | - | | 1.00 | 0.15 | - | 2.63 |
| Schmilau | 1,175 | <5 |  | 0.00 |  | - |  |  |  | 0.00 |  | - | | 1.01 | 0.15 | - | 2.67 |
| Schnakenbek | 1,857 | <5 |  | 0.00 |  | - |  |  |  | 0.00 |  | - | | 0.99 | 0.15 | - | 2.65 |
| Schönberg | 3,666 | <5 |  | 0.00 |  | - |  |  |  | 0.00 |  | - | | 0.96 | 0.14 | - | 2.55 |
| Schretstaken | 1,113 | <5 |  | 0.00 |  | - |  |  |  | 0.00 |  | - | | 1.01 | 0.15 | - | 2.64 |
| Schürensöhlen | 314 | <5 |  | 63.29 |  | - |  |  |  | 61.63 |  | - | | 1.48 | 0.33 | - | 3.47 |
| Schulendorf | 1,161 | <5 |  | 0.00 |  | - |  |  |  | 0.00 |  | - | | 1.01 | 0.15 | - | 2.71 |
| Schwarzenbek | 41,024 | <5 |  | 0.94 |  | - |  |  |  | 0.92 |  | - | | 0.99 | 0.29 | - | 2.10 |
| Seedorf | 936 | <5 |  | 0.00 |  | - |  |  |  | 0.00 |  | - | | 1.02 | 0.15 | - | 2.68 |
| Siebenbäumen | 1,765 | <5 |  | 0.00 |  | - |  |  |  | 0.00 |  | - | | 1.01 | 0.15 | - | 2.67 |
| Siebeneichen | 636 | <5 |  | 0.00 |  | - |  |  |  | 0.00 |  | - | | 1.04 | 0.16 | - | 2.71 |
| Sierksrade | 1,146 | <5 |  | 0.00 |  | - |  |  |  | 0.00 |  | - | | 1.01 | 0.15 | - | 2.72 |
| Sirksfelde | 735 | <5 |  | 25.67 |  | - |  |  |  | 25.07 |  | - | | 1.46 | 0.34 | - | 3.42 |
| Steinhorst | 1,417 | <5 |  | 0.00 |  | - |  |  |  | 0.00 |  | - | | 1.01 | 0.15 | - | 2.67 |
| Sterley | 2,469 | <5 |  | 0.00 |  | - |  |  |  | 0.00 |  | - | | 0.99 | 0.15 | - | 2.58 |
| Stubben | 908 | <5 |  | 0.00 |  | - |  |  |  | 0.00 |  | - | | 1.01 | 0.14 | - | 2.69 |
| Talkau | 1,144 | <5 |  | 0.00 |  | - |  |  |  | 0.00 |  | - | | 1.01 | 0.15 | - | 2.67 |
| Tramm | 912 | <5 |  | 0.00 |  | - |  |  |  | 0.00 |  | - | | 1.01 | 0.16 | - | 2.64 |
| Walksfelde | 521 | <5 |  | 0.00 |  | - |  |  |  | 0.00 |  | - | | 1.03 | 0.15 | - | 2.73 |
| Wangelau | 581 | <5 |  | 0.00 |  | - |  |  |  | 0.00 |  | - | | 1.03 | 0.16 | - | 2.74 |
| Wentorf bei Hamburg | 29,042 | <5 |  | 1.34 |  | - |  |  |  | 1.30 |  | - | | 1.15 | 0.33 | - | 2.47 |
| Wentorf (Amt Sandesneben) | 1,779 | <5 |  | 0.00 |  | - |  |  |  | 0.00 |  | - | | 1.00 | 0.15 | - | 2.64 |
| Wiershop | 383 | <5 |  | 0.00 |  | - |  |  |  | 0.00 |  | - | | 1.03 | 0.15 | - | 2.70 |
| Witzeeze | 1,951 | <5 |  | 0.00 |  | - |  |  |  | 0.00 |  | - | | 0.99 | 0.14 | - | 2.58 |
| Wohltorf | 5,665 | <5 |  | 0.00 |  | - |  |  |  | 0.00 |  | - | | 0.92 | 0.13 | - | 2.44 |
| Woltersdorf | 612 | <5 |  | 0.00 |  | - |  |  |  | 0.00 |  | - | | 1.02 | 0.15 | - | 2.67 |
| Worth | 396 | <5 |  | 0.00 |  | - |  |  |  | 0.00 |  | - | | 1.04 | 0.15 | - | 2.72 |
| Ziethen | 2,757 | <5 |  | 0.00 |  | - |  |  |  | 0.00 |  | - | | 0.98 | 0.15 | - | 2.59 |
| Bönningstedt | 11,617 | <5 |  | 0.00 |  | - |  |  |  | 0.00 |  | - | | 0.81 | 0.12 | - | 2.13 |
| Ellerbek | 9,221 | <5 |  | 0.00 |  | - |  |  |  | 0.00 |  | - | | 0.85 | 0.13 | - | 2.25 |
| Halstenbek | 38,263 | <5 |  | 0.50 |  | - |  |  |  | 0.49 |  | - | | 0.77 | 0.18 | - | 1.79 |
| Hasloh | 7,944 | <5 |  | 2.41 |  | - |  |  |  | 2.34 |  | - | | 1.24 | 0.29 | - | 2.91 |
| Pinneberg | 97,112 | <5 |  | 0.59 |  | - |  |  |  | 0.57 |  | - | | 0.71 | 0.24 | - | 1.43 |
| Rellingen | 29,275 | <5 |  | 1.97 |  | - |  |  |  | 1.91 |  | - | | 1.40 | 0.47 | - | 2.83 |
| Schenefeld | 37,854 | <5 |  | 0.51 |  | - |  |  |  | 0.50 |  | - | | 0.78 | 0.18 | - | 1.82 |
| Tangstedt | 5,582 | <5 |  | 0.00 |  | - |  |  |  | 0.00 |  | - | | 0.91 | 0.13 | - | 2.45 |
| Itzstedt | 6,093 | <5 |  | 0.00 |  | - |  |  |  | 0.00 |  | - | | 0.91 | 0.14 | - | 2.38 |
| Kayhude | 2,633 | <5 |  | 7.08 |  | - |  |  |  | 6.89 |  | - | | 1.40 | 0.32 | - | 3.29 |
| Nahe | 5,174 | <5 |  | 0.00 |  | - |  |  |  | 0.00 |  | - | | 0.93 | 0.14 | - | 2.43 |
| Norderstedt | 154,481 | 9 | 8.12 | 1.11 | 0.51 | - | 2.10 |  | 8.34 | 1.08 | 0.49 | - | 2.05 | 1.07 | 0.54 | - | 1.77 |
| Sülfeld | 7,600 | <5 |  | 0.00 |  | - |  |  |  | 0.00 |  | - | | 0.88 | 0.14 | - | 2.31 |
| Wakendorf II | 3,372 | <5 |  | 0.00 |  | - |  |  |  | 0.00 |  | - | | 0.97 | 0.15 | - | 2.55 |
| Ahrensburg | 73,007 | <5 |  | 1.06 |  | - |  |  |  | 1.03 |  | - | | 1.03 | 0.39 | - | 1.97 |
| Bad Oldesloe | 58,552 | <5 |  | 1.32 |  | - |  |  |  | 1.28 |  | - | | 1.19 | 0.44 | - | 2.27 |
| Bargfeld-Stegen | 8,339 | <5 |  | 2.37 |  | - |  |  |  | 2.30 |  | - | | 1.25 | 0.28 | - | 2.93 |
| Bargteheide | 39,985 | <5 |  | 0.98 |  | - |  |  |  | 0.95 |  | - | | 0.99 | 0.28 | - | 2.15 |
| Barnitz | 2,179 | <5 |  | 0.00 |  | - |  |  |  | 0.00 |  | - | | 0.99 | 0.14 | - | 2.60 |
| Barsbüttel | 26,469 | <5 |  | 1.47 |  | - |  |  |  | 1.43 |  | - | | 1.20 | 0.34 | - | 2.57 |
| Braak | 1,931 | <5 |  | 0.00 |  | - |  |  |  | 0.00 |  | - | | 1.00 | 0.15 | - | 2.68 |
| Delingsdorf | 6,761 | <5 |  | 0.00 |  | - |  |  |  | 0.00 |  | - | | 0.89 | 0.13 | - | 2.38 |
| Elmenhorst | 6,584 | <5 |  | 5.76 |  | - |  |  |  | 5.62 |  | - | | 1.67 | 0.49 | - | 3.59 |
| Glinde | 38,301 | <5 |  | 1.49 |  | - |  |  |  | 1.45 |  | - | | 1.24 | 0.41 | - | 2.49 |
| Grabau | 1,984 | <5 |  | 0.00 |  | - |  |  |  | 0.00 |  | - | | 0.99 | 0.15 | - | 2.63 |
| Grande | 1,289 | <5 |  | 0.00 |  | - |  |  |  | 0.00 |  | - | | 1.01 | 0.15 | - | 2.70 |
| Grönwohld | 3,104 | <5 |  | 0.00 |  | - |  |  |  | 0.00 |  | - | | 0.96 | 0.14 | - | 2.54 |
| Großensee | 3,701 | <5 |  | 5.13 |  | - |  |  |  | 4.98 |  | - | | 1.36 | 0.31 | - | 3.19 |
| Großhansdorf | 20,221 | <5 |  | 0.00 |  | - |  |  |  | 0.00 |  | - | | 0.70 | 0.10 | - | 1.84 |
| Hamfelde | 880 | <5 |  | 0.00 |  | - |  |  |  | 0.00 |  | - | | 1.02 | 0.15 | - | 2.71 |
| Hammoor | 3,382 | <5 |  | 0.00 |  | - |  |  |  | 0.00 |  | - | | 0.96 | 0.14 | - | 2.52 |
| Hohenfelde | 93 | <5 |  | 0.00 |  | - |  |  |  | 0.00 |  | - | | 1.03 | 0.15 | - | 2.70 |
| Hoisdorf | 8,386 | <5 |  | 0.00 |  | - |  |  |  | 0.00 |  | - | | 0.87 | 0.13 | - | 2.29 |
| Jersbek | 4,112 | <5 |  | 4.83 |  | - |  |  |  | 4.66 |  | - | | 1.35 | 0.32 | - | 3.13 |
| Klein Wesenberg | 1,967 | <5 |  | 0.00 |  | - |  |  |  | 0.00 |  | - | | 0.99 | 0.15 | - | 2.61 |
| Köthel | 562 | <5 |  | 0.00 |  | - |  |  |  | 0.00 |  | - | | 1.03 | 0.15 | - | 2.72 |
| Lütjensee | 8,127 | <5 |  | 0.00 |  | - |  |  |  | 0.00 |  | - | | 0.88 | 0.13 | - | 2.30 |
| Meddewade | 2,014 | <5 |  | 0.00 |  | - |  |  |  | 0.00 |  | - | | 0.99 | 0.15 | - | 2.61 |
| Neritz | 564 | <5 |  | 0.00 |  | - |  |  |  | 0.00 |  | - | | 1.02 | 0.15 | - | 2.68 |
| Nienwohld | 1,186 | <5 |  | 0.00 |  | - |  |  |  | 0.00 |  | - | | 1.01 | 0.15 | - | 2.68 |
| Oststeinbek | 16,382 | <5 |  | 0.00 |  | - |  |  |  | 0.00 |  | - | | 0.74 | 0.11 | - | 1.95 |
| Pölitz | 2,702 | <5 |  | 0.00 |  | - |  |  |  | 0.00 |  | - | | 0.98 | 0.15 | - | 2.62 |
| Rausdorf | 421 | <5 |  | 0.00 |  | - |  |  |  | 0.00 |  | - | | 1.04 | 0.15 | - | 2.75 |
| Reinbek | 57,782 | <5 |  | 1.00 |  | - |  |  |  | 0.97 |  | - | | 1.01 | 0.34 | - | 2.02 |
| Reinfeld (Holstein) | 20,634 | <5 |  | 0.95 |  | - |  |  |  | 0.92 |  | - | | 1.00 | 0.23 | - | 2.34 |
| Rethwisch | 2,856 | <5 |  | 0.00 |  | - |  |  |  | 0.00 |  | - | | 0.98 | 0.15 | - | 2.57 |
| Rümpel | 3,156 | <5 |  | 0.00 |  | - |  |  |  | 0.00 |  | - | | 0.97 | 0.14 | - | 2.57 |
| Siek | 5,512 | <5 |  | 3.48 |  | - |  |  |  | 3.36 |  | - | | 1.31 | 0.30 | - | 3.08 |
| Stapelfeld | 3,772 | <5 |  | 5.14 |  | - |  |  |  | 5.02 |  | - | | 1.38 | 0.32 | - | 3.22 |
| Tangstedt | 14,865 | <5 |  | 1.28 |  | - |  |  |  | 1.25 |  | - | | 1.09 | 0.24 | - | 2.57 |
| Todendorf | 2,695 | <5 |  | 0.00 |  | - |  |  |  | 0.00 |  | - | | 0.98 | 0.14 | - | 2.60 |
| Tremsbüttel | 5,195 | <5 |  | 0.00 |  | - |  |  |  | 0.00 |  | - | | 0.93 | 0.14 | - | 2.46 |
| Trittau | 19,306 | <5 |  | 1.02 |  | - |  |  |  | 0.99 |  | - | | 1.03 | 0.24 | - | 2.36 |
| Westerau | 1,826 | <5 |  | 0.00 |  | - |  |  |  | 0.00 |  | - | | 1.00 | 0.15 | - | 2.65 |
| Witzhave | 3,603 | <5 |  | 0.00 |  | - |  |  |  | 0.00 |  | - | | 0.96 | 0.15 | - | 2.52 |
| Brunsbek | 4,086 | <5 |  | 0.00 |  | - |  |  |  | 0.00 |  | - | | 0.96 | 0.14 | - | 2.54 |
| Lasbek | 2,861 | <5 |  | 0.00 |  | - |  |  |  | 0.00 |  | - | | 0.98 | 0.14 | - | 2.60 |
| Ammersbek | 21,872 | <5 |  | 0.88 |  | - |  |  |  | 0.86 |  | - | | 0.98 | 0.23 | - | 2.29 |
| Steinburg | 6,619 | <5 |  | 0.00 |  | - |  |  |  | 0.00 |  | - | | 0.89 | 0.14 | - | 2.36 |
| Travenbrück | 4,410 | <5 |  | 4.46 |  | - |  |  |  | 4.33 |  | - | | 1.34 | 0.31 | - | 3.13 |
| Wesenberg | 5,003 | <5 |  | 3.79 |  | - |  |  |  | 3.69 |  | - | | 1.34 | 0.30 | - | 3.09 |
| Hamburg | 3,786,828 | 219 | 203.67 | 1.08 | 0.94 | - | 1.23 |  | 208.42 | 1.05 | 0.92 | - | 1.20 | 1.05 | 0.92 | - | 1.20 |
| Appel | 3,954 | <5 |  | 4.97 |  | - |  |  |  | 4.82 |  | - | | 1.36 | 0.31 | - | 3.16 |
| Asendorf | 4,457 | <5 |  | 4.36 |  | - |  |  |  | 4.22 |  | - | | 1.34 | 0.30 | - | 3.13 |
| Bendestorf | 5,259 | <5 |  | 3.70 |  | - |  |  |  | 3.58 |  | - | | 1.32 | 0.30 | - | 3.11 |
| Brackel | 4,331 | <5 |  | 4.49 |  | - |  |  |  | 4.37 |  | - | | 1.35 | 0.31 | - | 3.17 |
| Buchholz in der Nordheide | 94,559 | <5 |  | 0.61 |  | - |  |  |  | 0.60 |  | - | | 0.73 | 0.25 | - | 1.48 |
| Dohren | 2,795 | <5 |  | 0.00 |  | - |  |  |  | 0.00 |  | - | | 0.97 | 0.14 | - | 2.55 |
| Drage | 10,206 | <5 |  | 0.00 |  | - |  |  |  | 0.00 |  | - | | 0.84 | 0.12 | - | 2.23 |
| Drestedt | 1,790 | <5 |  | 0.00 |  | - |  |  |  | 0.00 |  | - | | 1.00 | 0.15 | - | 2.62 |
| Egestorf | 5,761 | <5 |  | 0.00 |  | - |  |  |  | 0.00 |  | - | | 0.92 | 0.14 | - | 2.41 |
| Eyendorf | 3,246 | <5 |  | 0.00 |  | - |  |  |  | 0.00 |  | - | | 0.96 | 0.14 | - | 2.56 |
| Garlstorf | 2,575 | <5 |  | 7.41 |  | - |  |  |  | 7.20 |  | - | | 1.40 | 0.32 | - | 3.28 |
| Garstedt | 3,269 | <5 |  | 0.00 |  | - |  |  |  | 0.00 |  | - | | 0.96 | 0.14 | - | 2.53 |
| Gödenstorf | 2,429 | <5 |  | 8.13 |  | - |  |  |  | 7.84 |  | - | | 1.42 | 0.32 | - | 3.35 |
| Handeloh | 5,114 | <5 |  | 0.00 |  | - |  |  |  | 0.00 |  | - | | 0.93 | 0.14 | - | 2.43 |
| Hanstedt | 11,357 | <5 |  | 6.85 |  | - |  |  |  | 6.64 |  | - | | 2.23 | 0.83 | - | 4.33 |
| Harmstorf | 2,277 | <5 |  | 0.00 |  | - |  |  |  | 0.00 |  | - | | 0.99 | 0.15 | - | 2.65 |
| Hollenstedt | 9,171 | <5 |  | 0.00 |  | - |  |  |  | 0.00 |  | - | | 0.85 | 0.13 | - | 2.24 |
| Jesteburg | 17,869 | <5 |  | 1.09 |  | - |  |  |  | 1.06 |  | - | | 1.04 | 0.23 | - | 2.43 |
| Kakenstorf | 3,333 | <5 |  | 0.00 |  | - |  |  |  | 0.00 |  | - | | 0.96 | 0.15 | - | 2.57 |
| Marschacht | 9,077 | <5 |  | 0.00 |  | - |  |  |  | 0.00 |  | - | | 0.85 | 0.13 | - | 2.23 |
| Marxen | 3,520 | <5 |  | 0.00 |  | - |  |  |  | 0.00 |  | - | | 0.96 | 0.14 | - | 2.56 |
| Moisburg | 4,627 | <5 |  | 0.00 |  | - |  |  |  | 0.00 |  | - | | 0.94 | 0.14 | - | 2.50 |
| Neu Wulmstorf | 49,421 | <5 |  | 1.19 |  | - |  |  |  | 1.15 |  | - | | 1.11 | 0.37 | - | 2.22 |
| Otter | 4,301 | <5 |  | 0.00 |  | - |  |  |  | 0.00 |  | - | | 0.95 | 0.14 | - | 2.52 |
| Rosengarten | 31,723 | <5 |  | 0.61 |  | - |  |  |  | 0.59 |  | - | | 0.85 | 0.19 | - | 1.96 |
| Salzhausen | 12,004 | <5 |  | 1.65 |  | - |  |  |  | 1.60 |  | - | | 1.17 | 0.27 | - | 2.74 |
| Seevetal | 89,440 | <5 |  | 0.22 |  | - |  |  |  | 0.21 |  | - | | 0.48 | 0.11 | - | 1.09 |
| Stelle | 25,220 | <5 |  | 0.00 |  | - |  |  |  | 0.00 |  | - | | 0.65 | 0.10 | - | 1.72 |
| Tespe | 7,790 | <5 |  | 0.00 |  | - |  |  |  | 0.00 |  | - | | 0.88 | 0.13 | - | 2.31 |
| Toppenstedt | 4,860 | <5 |  | 4.06 |  | - |  |  |  | 3.93 |  | - | | 1.34 | 0.30 | - | 3.14 |
| Tostedt | 33,399 | <5 |  | 0.00 |  | - |  |  |  | 0.00 |  | - | | 0.58 | 0.09 | - | 1.53 |
| Undeloh | 1,844 | <5 |  | 0.00 |  | - |  |  |  | 0.00 |  | - | | 0.99 | 0.15 | - | 2.62 |
| Vierhöfen | 2,457 | <5 |  | 0.00 |  | - |  |  |  | 0.00 |  | - | | 0.98 | 0.15 | - | 2.58 |
| Welle | 3,182 | <5 |  | 0.00 |  | - |  |  |  | 0.00 |  | - | | 0.97 | 0.14 | - | 2.54 |
| Wenzendorf | 3,167 | <5 |  | 6.10 |  | - |  |  |  | 5.91 |  | - | | 1.38 | 0.32 | - | 3.21 |
| Winsen (Luhe) | 84,737 | 7 | 4.44 | 1.58 | 0.63 | - | 3.25 |  | 4.56 | 1.54 | 0.62 | - | 3.16 | 1.37 | 0.64 | - | 2.37 |
| Wulfsen | 4,261 | <5 |  | 0.00 |  | - |  |  |  | 0.00 |  | - | | 0.94 | 0.14 | - | 2.47 |
| Göhrde | 1,269 | <5 |  | 0.00 |  | - |  |  |  | 0.00 |  | - | | 1.00 | 0.14 | - | 2.58 |
| Neu Darchau | 2,937 | <5 |  | 0.00 |  | - |  |  |  | 0.00 |  | - | | 0.97 | 0.14 | - | 2.58 |
| Adendorf | 24,075 | <5 |  | 1.61 |  | - |  |  |  | 1.56 |  | - | | 1.23 | 0.36 | - | 2.63 |
| Amelinghausen | 10,077 | <5 |  | 0.00 |  | - |  |  |  | 0.00 |  | - | | 0.84 | 0.13 | - | 2.21 |
| Artlenburg | 4,201 | <5 |  | 0.00 |  | - |  |  |  | 0.00 |  | - | | 0.95 | 0.14 | - | 2.47 |
| Bardowick | 17,283 | <5 |  | 0.00 |  | - |  |  |  | 0.00 |  | - | | 0.75 | 0.11 | - | 1.94 |
| Barendorf | 7,788 | <5 |  | 2.48 |  | - |  |  |  | 2.41 |  | - | | 1.24 | 0.28 | - | 2.90 |
| Barnstedt | 1,719 | <5 |  | 0.00 |  | - |  |  |  | 0.00 |  | - | | 1.01 | 0.15 | - | 2.66 |
| Barum | 4,808 | <5 |  | 8.09 |  | - |  |  |  | 7.84 |  | - | | 1.75 | 0.51 | - | 3.78 |
| Betzendorf | 3,017 | <5 |  | 0.00 |  | - |  |  |  | 0.00 |  | - | | 0.97 | 0.14 | - | 2.58 |
| Bleckede | 22,826 | <5 |  | 0.88 |  | - |  |  |  | 0.85 |  | - | | 0.98 | 0.22 | - | 2.27 |
| Boitze | 837 | <5 |  | 0.00 |  | - |  |  |  | 0.00 |  | - | | 1.02 | 0.15 | - | 2.67 |
| Brietlingen | 9,594 | <5 |  | 6.10 |  | - |  |  |  | 5.93 |  | - | | 1.95 | 0.66 | - | 3.95 |
| Dahlem | 1,118 | <5 |  | 0.00 |  | - |  |  |  | 0.00 |  | - | | 1.01 | 0.16 | - | 2.68 |
| Dahlenburg | 8,382 | <5 |  | 0.00 |  | - |  |  |  | 0.00 |  | - | | 0.87 | 0.13 | - | 2.31 |
| Deutsch Evern | 9,383 | <5 |  | 2.08 |  | - |  |  |  | 2.02 |  | - | | 1.22 | 0.30 | - | 2.84 |
| Echem | 3,008 | <5 |  | 0.00 |  | - |  |  |  | 0.00 |  | - | | 0.98 | 0.15 | - | 2.55 |
| Embsen | 7,094 | <5 |  | 0.00 |  | - |  |  |  | 0.00 |  | - | | 0.89 | 0.14 | - | 2.34 |
| Handorf | 5,461 | <5 |  | 0.00 |  | - |  |  |  | 0.00 |  | - | | 0.92 | 0.13 | - | 2.42 |
| Hittbergen | 2,175 | <5 |  | 0.00 |  | - |  |  |  | 0.00 |  | - | | 0.99 | 0.15 | - | 2.62 |
| Hohnstorf (Elbe) | 5,621 | <5 |  | 3.62 |  | - |  |  |  | 3.49 |  | - | | 1.32 | 0.30 | - | 3.08 |
| Kirchgellersen | 6,106 | <5 |  | 3.09 |  | - |  |  |  | 3.01 |  | - | | 1.30 | 0.29 | - | 3.03 |
| Lüdersburg | 1,394 | <5 |  | 0.00 |  | - |  |  |  | 0.00 |  | - | | 1.01 | 0.15 | - | 2.68 |
| Lüneburg | 157,059 | 10 | 8.34 | 1.20 | 0.58 | - | 2.21 |  | 8.55 | 1.17 | 0.56 | - | 2.15 | 1.14 | 0.60 | - | 1.84 |
| Mechtersen | 1,548 | <5 |  | 0.00 |  | - |  |  |  | 0.00 |  | - | | 1.01 | 0.15 | - | 2.63 |
| Melbeck | 8,465 | <5 |  | 4.72 |  | - |  |  |  | 4.56 |  | - | | 1.61 | 0.46 | - | 3.46 |
| Nahrendorf | 3,206 | <5 |  | 6.18 |  | - |  |  |  | 5.96 |  | - | | 1.38 | 0.32 | - | 3.26 |
| Neetze | 6,964 | <5 |  | 0.00 |  | - |  |  |  | 0.00 |  | - | | 0.90 | 0.14 | - | 2.37 |
| Oldendorf (Luhe) | 2,323 | <5 |  | 0.00 |  | - |  |  |  | 0.00 |  | - | | 0.98 | 0.15 | - | 2.62 |
| Radbruch | 5,210 | <5 |  | 0.00 |  | - |  |  |  | 0.00 |  | - | | 0.92 | 0.14 | - | 2.40 |
| Rehlingen | 1,526 | <5 |  | 12.82 |  | - |  |  |  | 12.46 |  | - | | 1.44 | 0.33 | - | 3.39 |
| Reinstorf | 3,250 | <5 |  | 0.00 |  | - |  |  |  | 0.00 |  | - | | 0.97 | 0.14 | - | 2.55 |
| Reppenstedt | 17,250 | <5 |  | 1.13 |  | - |  |  |  | 1.10 |  | - | | 1.06 | 0.24 | - | 2.44 |
| Rullstorf | 5,081 | <5 |  | 0.00 |  | - |  |  |  | 0.00 |  | - | | 0.94 | 0.14 | - | 2.46 |
| Scharnebeck | 8,969 | <5 |  | 0.00 |  | - |  |  |  | 0.00 |  | - | | 0.86 | 0.13 | - | 2.25 |
| Soderstorf | 3,789 | <5 |  | 0.00 |  | - |  |  |  | 0.00 |  | - | | 0.95 | 0.14 | - | 2.56 |
| Südergellersen | 4,222 | <5 |  | 0.00 |  | - |  |  |  | 0.00 |  | - | | 0.95 | 0.14 | - | 2.49 |
| Thomasburg | 3,483 | <5 |  | 0.00 |  | - |  |  |  | 0.00 |  | - | | 0.95 | 0.13 | - | 2.53 |
| Tosterglope | 1,124 | <5 |  | 0.00 |  | - |  |  |  | 0.00 |  | - | | 1.01 | 0.15 | - | 2.67 |
| Vastorf | 1,872 | <5 |  | 0.00 |  | - |  |  |  | 0.00 |  | - | | 1.00 | 0.15 | - | 2.63 |
| Vögelsen | 5,512 | <5 |  | 0.00 |  | - |  |  |  | 0.00 |  | - | | 0.92 | 0.14 | - | 2.41 |
| Wendisch Evern | 4,448 | <5 |  | 0.00 |  | - |  |  |  | 0.00 |  | - | | 0.94 | 0.14 | - | 2.48 |
| Westergellersen | 5,371 | <5 |  | 0.00 |  | - |  |  |  | 0.00 |  | - | | 0.93 | 0.14 | - | 2.45 |
| Wittorf | 3,845 | <5 |  | 5.08 |  | - |  |  |  | 4.90 |  | - | | 1.36 | 0.31 | - | 3.19 |
| Amt Neuhaus | 9,526 | <5 |  | 0.00 |  | - |  |  |  | 0.00 |  | - | | 0.85 | 0.12 | - | 2.23 |
| Bispingen | 15,013 | <5 |  | 1.31 |  | - |  |  |  | 1.27 |  | - | | 1.11 | 0.25 | - | 2.59 |
| Altenmedingen | 3,614 | <5 |  | 0.00 |  | - |  |  |  | 0.00 |  | - | | 0.97 | 0.15 | - | 2.56 |
| Bad Bevensen | 14,249 | <5 |  | 1.39 |  | - |  |  |  | 1.35 |  | - | | 1.12 | 0.26 | - | 2.61 |
| Barum | 1,594 | <5 |  | 0.00 |  | - |  |  |  | 0.00 |  | - | | 1.00 | 0.15 | - | 2.66 |
| Bienenbüttel | 16,276 | <5 |  | 0.00 |  | - |  |  |  | 0.00 |  | - | | 0.75 | 0.11 | - | 1.97 |
| Ebstorf | 13,103 | <5 |  | 0.00 |  | - |  |  |  | 0.00 |  | - | | 0.80 | 0.12 | - | 2.13 |
| Emmendorf | 1,358 | <5 |  | 0.00 |  | - |  |  |  | 0.00 |  | - | | 1.01 | 0.15 | - | 2.69 |
| Hanstedt | 1,916 | <5 |  | 0.00 |  | - |  |  |  | 0.00 |  | - | | 1.01 | 0.15 | - | 2.67 |
| Himbergen | 3,667 | <5 |  | 0.00 |  | - |  |  |  | 0.00 |  | - | | 0.97 | 0.14 | - | 2.56 |
| Jelmstorf | 1,357 | <5 |  | 0.00 |  | - |  |  |  | 0.00 |  | - | | 1.01 | 0.15 | - | 2.64 |
| Natendorf | 2,048 | <5 |  | 10.03 |  | - |  |  |  | 9.70 |  | - | | 1.42 | 0.33 | - | 3.32 |
| Oetzen | 2,873 | <5 |  | 0.00 |  | - |  |  |  | 0.00 |  | - | | 0.97 | 0.15 | - | 2.54 |
| Römstedt | 1,747 | <5 |  | 0.00 |  | - |  |  |  | 0.00 |  | - | | 1.00 | 0.15 | - | 2.61 |
| Schwienau | 1,701 | <5 |  | 0.00 |  | - |  |  |  | 0.00 |  | - | | 1.01 | 0.14 | - | 2.67 |
| Stoetze | 1,403 | <5 |  | 0.00 |  | - |  |  |  | 0.00 |  | - | | 1.01 | 0.15 | - | 2.68 |
| Weste | 2,319 | <5 |  | 8.80 |  | - |  |  |  | 8.49 |  | - | | 1.42 | 0.33 | - | 3.30 |
| Wriedel | 5,623 | <5 |  | 0.00 |  | - |  |  |  | 0.00 |  | - | | 0.92 | 0.14 | - | 2.42 |
| Dechow | 554 | <5 |  | 0.00 |  | - |  |  |  | 0.00 |  | - | | 1.03 | 0.16 | - | 2.76 |
| Groß Molzahn | 907 | <5 |  | 0.00 |  | - |  |  |  | 0.00 |  | - | | 1.02 | 0.15 | - | 2.69 |
| Kneese | 727 | <5 |  | 0.00 |  | - |  |  |  | 0.00 |  | - | | 1.02 | 0.15 | - | 2.71 |
| Rögnitz | 330 | <5 |  | 0.00 |  | - |  |  |  | 0.00 |  | - | | 1.03 | 0.15 | - | 2.72 |
| Schlagsdorf | 2,844 | <5 |  | 0.00 |  | - |  |  |  | 0.00 |  | - | | 0.97 | 0.14 | - | 2.57 |
| Thandorf | 401 | <5 |  | 0.00 |  | - |  |  |  | 0.00 |  | - | | 1.03 | 0.15 | - | 2.70 |
| Utecht | 980 | <5 |  | 0.00 |  | - |  |  |  | 0.00 |  | - | | 1.02 | 0.15 | - | 2.70 |
| Bengerstorf | 1,531 | <5 |  | 0.00 |  | - |  |  |  | 0.00 |  | - | | 1.00 | 0.14 | - | 2.61 |
| Besitz | 731 | <5 |  | 0.00 |  | - |  |  |  | 0.00 |  | - | | 1.03 | 0.15 | - | 2.72 |
| Boizenburg/Elbe | 19,694 | <5 |  | 0.00 |  | - |  |  |  | 0.00 |  | - | | 0.70 | 0.11 | - | 1.87 |
| Brahlstorf | 1,396 | <5 |  | 0.00 |  | - |  |  |  | 0.00 |  | - | | 1.01 | 0.15 | - | 2.67 |
| Dersenow | 1,078 | <5 |  | 0.00 |  | - |  |  |  | 0.00 |  | - | | 1.02 | 0.15 | - | 2.66 |
| Gallin | 1,268 | <5 |  | 0.00 |  | - |  |  |  | 0.00 |  | - | | 1.01 | 0.14 | - | 2.68 |
| Gresse | 1,281 | <5 |  | 0.00 |  | - |  |  |  | 0.00 |  | - | | 1.00 | 0.15 | - | 2.64 |
| Greven | 1,414 | <5 |  | 0.00 |  | - |  |  |  | 0.00 |  | - | | 1.01 | 0.15 | - | 2.66 |
| Kogel | 1,458 | <5 |  | 0.00 |  | - |  |  |  | 0.00 |  | - | | 1.00 | 0.15 | - | 2.65 |
| Lübtheen | 9,227 | <5 |  | 0.00 |  | - |  |  |  | 0.00 |  | - | | 0.86 | 0.13 | - | 2.25 |
| Lüttow-Valluhn | 2,213 | <5 |  | 0.00 |  | - |  |  |  | 0.00 |  | - | | 0.99 | 0.15 | - | 2.61 |
| Neu Gülze | 1,809 | <5 |  | 0.00 |  | - |  |  |  | 0.00 |  | - | | 1.00 | 0.15 | - | 2.64 |
| Nostorf | 3,037 | <5 |  | 0.00 |  | - |  |  |  | 0.00 |  | - | | 0.96 | 0.15 | - | 2.58 |
| Pritzier | 875 | <5 |  | 0.00 |  | - |  |  |  | 0.00 |  | - | | 1.02 | 0.15 | - | 2.67 |
| Schwanheide | 1,510 | <5 |  | 0.00 |  | - |  |  |  | 0.00 |  | - | | 1.00 | 0.15 | - | 2.66 |
| Teldau | 2,071 | <5 |  | 0.00 |  | - |  |  |  | 0.00 |  | - | | 0.98 | 0.15 | - | 2.59 |
| Tessin b. Boizenburg | 1,020 | <5 |  | 0.00 |  | - |  |  |  | 0.00 |  | - | | 1.02 | 0.15 | - | 2.67 |
| Vellahn | 6,048 | <5 |  | 3.20 |  | - |  |  |  | 3.10 |  | - | | 1.31 | 0.30 | - | 3.08 |
| Warlitz | 1,001 | <5 |  | 0.00 |  | - |  |  |  | 0.00 |  | - | | 1.02 | 0.16 | - | 2.70 |
| Wittenburg | 13,144 | <5 |  | 0.00 |  | - |  |  |  | 0.00 |  | - | | 0.78 | 0.12 | - | 2.07 |
| Zarrentin am Schaalsee | 11,473 | <5 |  | 0.00 |  | - |  |  |  | 0.00 |  | - | | 0.82 | 0.12 | - | 2.18 |
| Toddin | 1,845 | <5 |  | 0.00 |  | - |  |  |  | 0.00 |  | - | | 1.00 | 0.14 | - | 2.65 |
|  |  |  |  |  |  |  |  |  |  |  |  |  |  |  |  |  |  |
| * To protect personal integrity and in compliance with the General Data Protection Regulation (GDPR) of the European Union and the German Childhood Cancer Registry’s informed consent procedure, we do not provide the exact number of observed and expected cases, and confidence intervals but use "<5" to denote subgroups including less than five observed childhood cancer cases. | | | | | | | | | | | | | | | | | |
